# Surgical Management of Musculoskeletal Injuries after 2015 Nepal Earthquake: Our Experience

**DOI:** 10.7759/cureus.306

**Published:** 2015-08-18

**Authors:** Raju Vaishya, Amit Kumar Agarwal, Vipul Vijay, Mustafa Hussaini, Harsh Singh

**Affiliations:** 1 Orthopaedics, Indraprastha Apollo Hospital; 2 Orthopaedics, Indraprastha Apollo Hospitals

**Keywords:** earthquake, disaster, orthopaedics, musculoskeletal injuries, fractures, nepal

## Abstract

We report our experience of handling 80 major musculoskeletal injuries in a brief span of three days immediately after the major earthquake of Nepal in April 2015. Planning, proper utilization of resources, and prioritizing the patients for surgical intervention is highlighted. The value of damage control by orthopaedics in these disasters is discussed. Timely and appropriate surgical treatment by a skilled orthopaedic team not only can save these injured limbs but also the lives of the victims of a major disaster.

## Introduction

**Figure 1:**
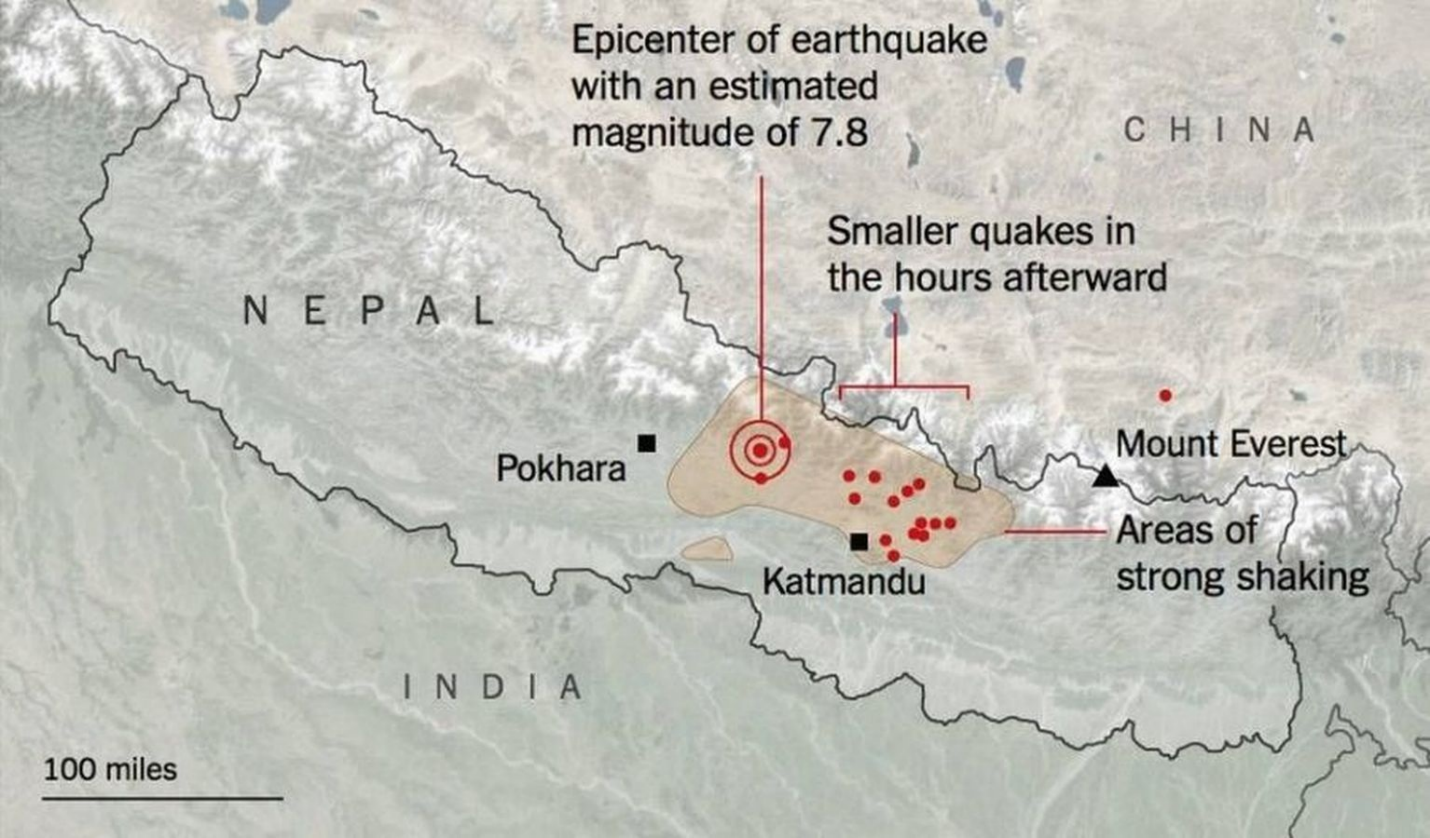
A map showing the epicenter and affected area of Nepal in the 2015 earthquake.

On April 25, 2015, an earthquake measuring 7.8 on the Richter scale struck Nepal with its epicenter at the Barpak Village in the Gorkha district and its hypocenter was at a depth of approximately 15 km (Figure 1).

**Figure 2:**
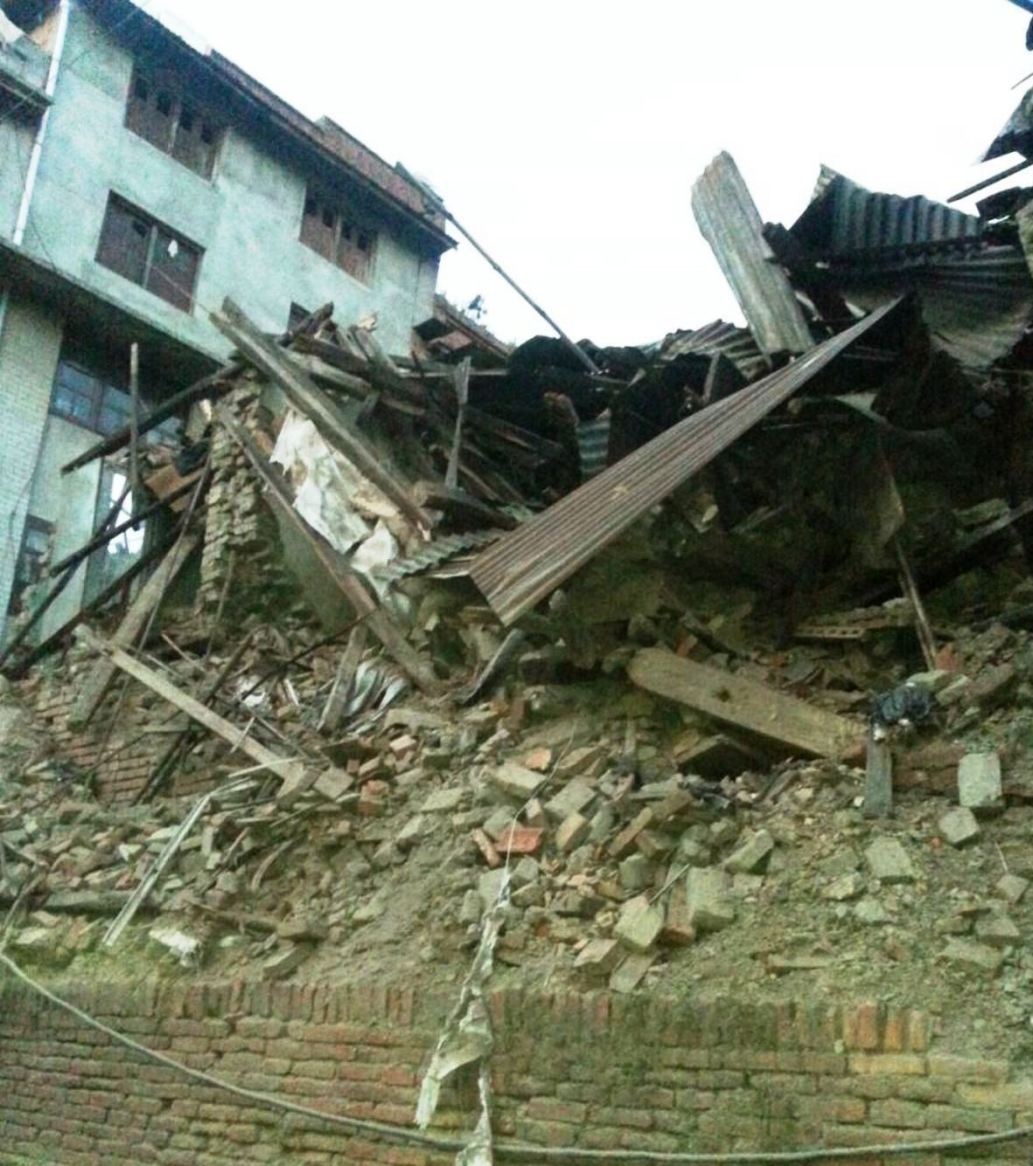
Severely damaged and collapsed buildings mainly responsible for injuries.

**Figure 3:**
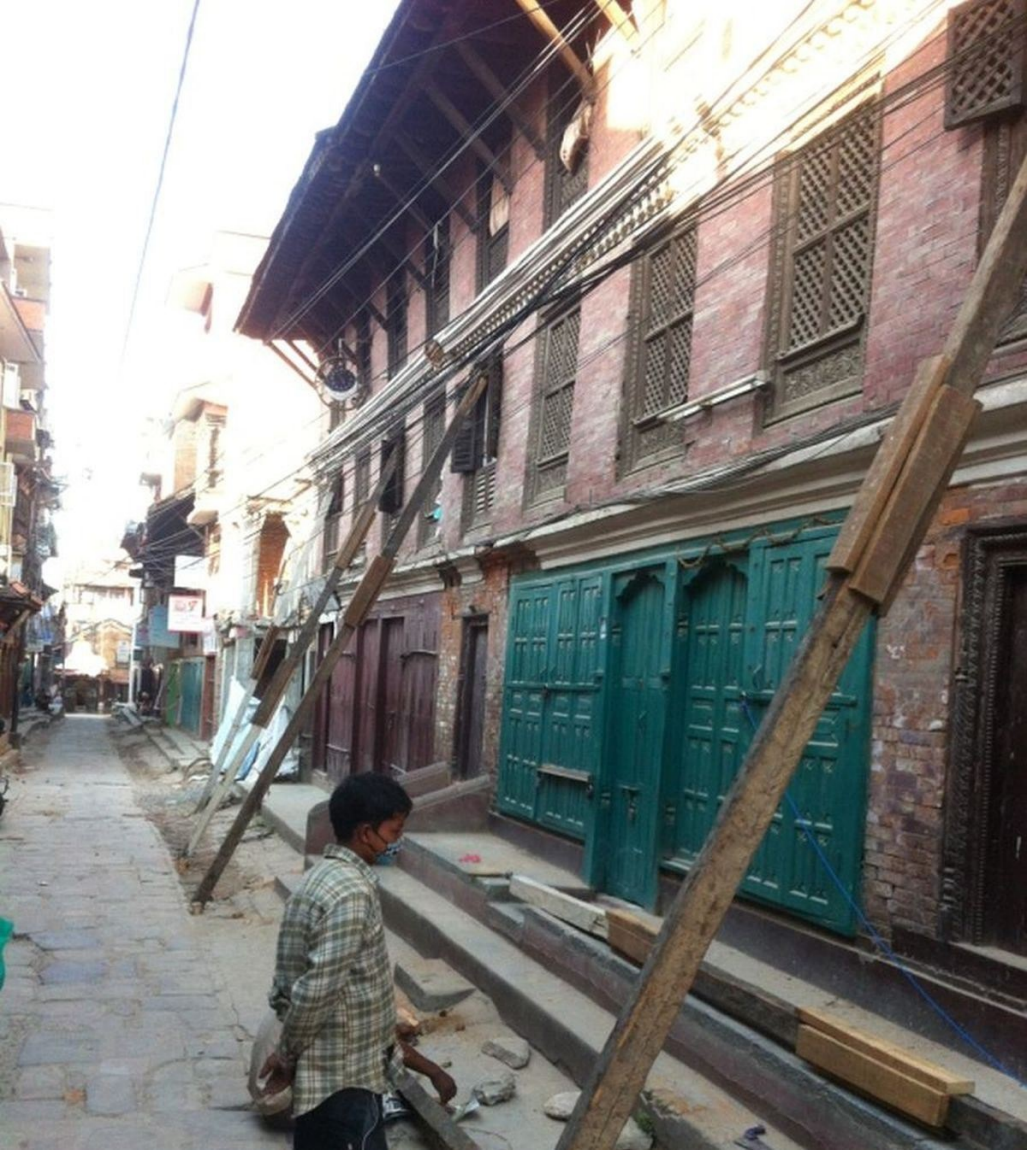
Damaged archeological monuments

There were more than 8,000 deaths and more than 14,000 people were injured. This earthquake triggered an avalanche on Mount Everest killing at least 19 mountaineers also. Thousands of people were made homeless across many districts of the country. Many archaeological monuments and centuries-old buildings were destroyed (Figures 2-3). Many buildings were ready to collapse due to earthquake-induced cracks, and these buildings were supported by logs of wood temporarily.

Earthquakes usually inflict more injuries and morbidity than mortality and these injuries are predominantly musculoskeletal in nature [1]. Initial management at the time of an earthquake is often hampered by the destruction of basic infrastructures and hospital facilities due to shocks [2]. Due to the sudden, unexpected, and disproportionate increase in the number of trauma patients as well as limited surgical and anesthetic support, often early definite orthopaedic management is usually not possible. The role of orthopaedic surgeons during rescue missions in such situations is, therefore, important [3]. We report the injury pattern and profile of musculoskeletal injuries managed by our team soon after the 2015 Nepal earthquake, within a short span of three days.

## Case presentation

Signed informed patient consent was waived due to circumstances of the emergency. No identify patient information was included in this report.

**Figure 4:**
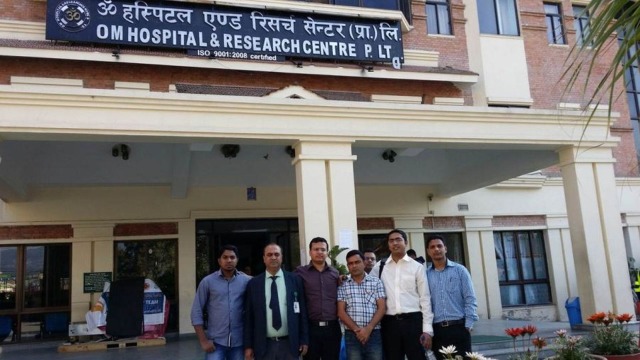
Rescue team members in front of local hospital at Kathmandu

Eighty earthquake victims with orthopaedic injuries were treated by a team of six medical staff at Om Hospital, Kathmandu (Figure 4).

Of the 80 patients treated, 46 were male and 34 were female patients aged between 6 and 96 (mean: 48) years. Their injury patterns, anesthesia administered, surgeries undertaken, and postoperative complications were analyzed. Almost all the major bones were found to be Injured in our series, including spine fractures. In our observation, a total of 56 patients had closed injuries managed with open reduction and internal fixation (ORIF) whereas unfortunately four patients ended up with an amputation of their limb.

### Orthopaedic team

**Figure 5:**
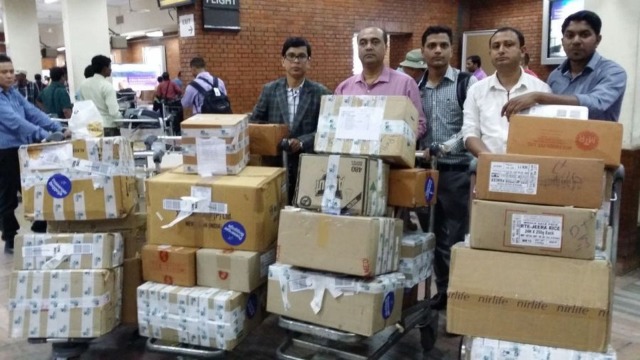
The rescue team arriving with medical supplies

After the news of massive earthquake affecting mass casualty in the Kathmandu valley, a rescue team was formed by our hospital and was sent immediately (after three days) to Kathmandu, Nepal. It comprised of three orthopaedic surgeons, one orthopaedic operating room (OR) nurse, and one general nurse. The team was deployed to a partially damaged operating theatre of one of the 250-bed private hospitals in Kathmandu, which had an emergency department and working operating theatre facilities. Our team brought in 800 kilograms of surgical, medical, and anaesthetic equipment from India for proper working in OR and management of injured patients (Figure 5). Our inventory included power drills, large/small fragment bone plate sets, wiring sets, external fixators, and intramedullary nails.

All 1,300 patients were first managed at the emergency department. The patients with minor injuries were treated (e.g, repair of lacerations, casts for non-displaced and minor fractures, etc.) by the emergency medical staff. Only those patients (80) with significant limb and spinal injuries were referred to our team for surgical management. All the patients who required surgical intervention underwent necessary preoperative laboratory and radiological investigations. Compound and crush injuries were given the priority over the closed injuries. The preoperative planning of all the cases was done prior to surgery on a ward round with the local medical team. We had adopted the principle of orthopaedic damage control for the treatment of these victims, whereby the emphasis was given to treat as many people as possible with available medical resources and to achieve maximum gains by doing minimum interventions.

### Surgery

**Figure 6:**
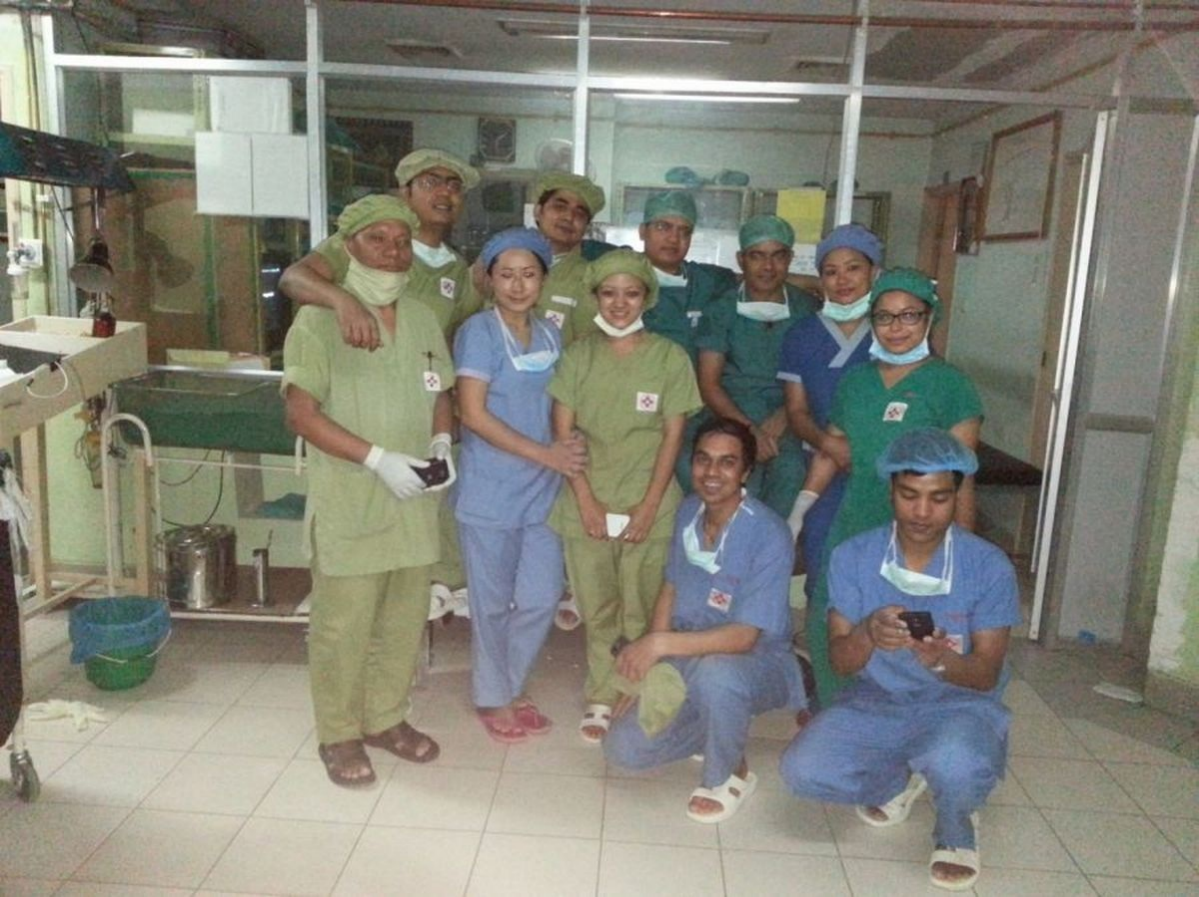
A combined force of local and guest surgical team members

We had two designated ORs for treating these 80 patients with extremity and spine injuries. These ORs functioned almost continuously for more than 12 hours daily for three days. Our team worked in association with the local staff of the hospital (Figure 6). All the patients were given a single preoperative prophylactic dose of intravenous broad spectrum antibiotic. Open fractures and crush injuries were thoroughly irrigated and washed with sterile saline, debridement of devitalized tissue was done, and the fractures were fixed using external fixators. For closed fractures with good skin and soft-tissue conditions, open reduction and internal fixation using a plate, nail, or wires were performed.

The injuries treated included that of the upper limbs, lower limbs, and spine. Almost all the major bones of the body were injured in different patients and were fixed accordingly. Out of nine patients with open injuries; five patients had a Gustilo Anderson Grade IIIA injury, three patients had a Grade IIIB injury, and one patient had a Grade II injury. Out of these nine patients, eight patients had lower limb injuries whereas only one patient had an upper limb injury. All these open injuries were managed by debridement and external fixation. We tried our best to avoid any amputation, but four patients had badly mangled extremities that could not be saved in spite of our best efforts. Out of these four patients; two patients had below the knee amputation, one patient had above the knee amputation, and one patient had a below the elbow amputation (Table 1).

**Table 1:** Details of the cases and management done during the rescue mission (ORIF: Open Reduction and Internal Fixation, CRIF: Closed Reduction and Internal Fixation, Ex Fix: External Fixator, K-Wire: Kirschner wire, K nail: Kuntscher nail, DHS: Dynamic Hip Screw, CCS: Cannulated Cancellous Screw)

Serial number	Diagnosis	Number of Cases	Treatment
1	Burst Fracture Spine	2	Pedicle screw fix. + decompression
2	Proximal Humeral Fracture	3	ORIF/CRIF + k-WIRE Fix
3	Humeral Shaft Fracture	3	ORIF
4	Distal Humeral Fracture	3	ORIF
5	Both Bones Forearm Fracture	6	ORIF
6	Distal Radius Fracture	4	ORIF/CRIF + k-WIRE Fix
7	Hand Fracture	2	CRIF + k-WIRE Fix
8	Fracture Neck of Femur	3	CRIF/Hemiarthroplasty
9	Intertrochanteric Fracture Femur	4	CRIF with DHS
10	Fracture Shaft Femur	6	ORIF with K-Nail/Plating
11	Fracture Distal Femur	4	ORIF
12	Proximal Tibia Fracture	3	ORIF
13	Tibial Shaft Fracture	9	ORIF with K-Nail/Plating
14	Distal Tibial Fracture	2	ORIF
15	Calcaneal Fracture	2	CRIF with CCS
16	Open Injuries	9	Deb + Ex Fix
17	Infected Wounds/Crush injury	15	Debridement/ Amputation
	Total No. of Cases	80	

### Postoperative care

All the open wounds were inspected every second day and repeated debridement was done in three patients with crush injuries. Patients were mobilized as early as possible after the surgery. Postoperative care and follow-up were continued by the local orthopaedic surgeons. We did not encounter any major surgical complication, neither lost any limb nor life of our operated patients during this rescue mission.

## Discussion

Asia witnessed many major earthquakes in the recent times: India in 2001, Iran in 2003, the Indian Ocean in 2004, Pakistan in 2005, China in 2008, and now Nepal in 2015 [4]. These natural calamities cause an enormous loss of life and economy [5]. When the Nepal earthquake struck in April 2015, there was a rush of rescue and medical help from various countries all over the world. As a real neighbor, we also decided to send our team from India to Nepal for rescue. During earthquakes, most injuries are orthopaedics related as they are caused mainly due to fall of buildings [6]. Our study also showed that the number of musculoskeletal injuries is higher than that of other systems as justified by the emergency room (ER) data. Various studies have shown that the numbers of casualties seen are directly proportionate to the density of a population in a particular region [7]. There is a high initial load of cases from the adjoining areas followed by a second wave of cases from the most peripheral areas as rescue operations reach those areas [8].

This Nepal earthquake occurred in the afternoon of a weekly holiday and the most victims were injured while escaping falling debris from the buildings [9]. Crush injuries of the limb in our series were not as common as in reports from other studies [10]. We believe that as the most houses were old and dilapidated and were not earthquake proof. Hence, these structures were unable to withstand the shocks and aftershocks of this massive earthquake. Many victims remained buried under debris because of delay of trained manpower and equipment to reach at ground zero which was located almost one hundred kilometers from Kathmandu, the capital city of Nepal. We noticed a trend of closed injuries more, and we presume that the patients with more severe compound and crush injuries had either died before they could be rescued from the site or they could not manage to reach the hospital. There is another possibility that our team was located 100 km from the epicentre and the scale of damage in the city itself was not as severe as elsewhere. There is always a delay in disaster response owing to the difficulties in reaching the epicenter due to loss of communication and transportation problems. Usually at the time of such a massive destruction and shortage of resources, the principles of orthopaedics damage control are followed [11]. Often in these situations, there is a lack of equipment, implants, and skilled manpower to perform challenging procedures, such as internal fixation of fractures. Because of the sudden rise in the demands of the orthopaedic implants and the lack of supplies from the distributors, local hospitals were running short of the implants. Our team consisted of three orthopaedic surgeons and two trained nursing staff, and we were carrying 800 kgs of medical and surgical items from India (including orthopaedic implants and instruments). This timely arrival of the orthopaedic surgical team with much-needed implants and tools to the local hospital within three days of the earthquake helped to manage the cases in timely and efficient manner. Ongoing aftershocks, the risks of infection, and the lack of proper food were a few of the challenges faced by our team in the aftermath of earthquakes in Nepal.

Such humanitarian rescue missions require dedicated and disciplined team members with high morale who can perform in these hostile conditions. It is difficult to maintain the mental and physical strength of the team for a prolonged period. Our team members were also involved in the training of the theatre and medical staff of the host hospital in Kathmandu. Local doctors were instrumental in supporting us by managing the patients in emergency and triaging them. This association between visiting and local doctors resulted in better and more timely management of the patients during this difficult time. Importance of cooperation and coordination between the local and foreign medical teams cannot be overemphasized. The proper coordination between medical and non-medical personnel from other countries helps achieve a better control of the situation. The coordination of our team with the local doctors and with the parent institute back home was crucial in the management of these causalities. We believe that the success of any rescue health mission depends not only on the level of preparedness but also depends on collaboration with local health care providers.

The foreign humanitarian and medical aid plays a pivotal role in natural disasters like an earthquake. Based on the severity of the catastrophe and the phase of disaster response, the epidemiology of the various medical problems may change accordingly. Orthopaedics remains a major subspecialty immediately after an earthquake as most of the patients will have musculoskeletal injuries. Study and research in this particular area and literature on disaster-related injury patterns and disaster-specific problems would help in improvising the response required in future in similar conditions [12-13]. The evidence-based research for disaster management helps in reducing the impact of disasters on the affected population. This mission aptly proved that "Great is the man with a great heart, not with great caste, creed, or birth." Although this rescue mission was like tiny drops in this massive disaster, little drops of water do make a mighty ocean!!!

## Conclusions

Foreign humanitarian and medical aid plays a pivotal role in natural disasters, such as described in the earthquake discussed in this paper. Orthopaedics remains a major subspecialty needed immediately after an earthquake as most of the patients have musculoskeletal injuries. Proper planning, timely intervention, utilization of resources, and surgical intervention by a skilled orthopaedic team can make a huge difference in the morbidity of patients in the wake of a major disaster.
